# Efficacy and Safety of the Combination of Durvalumab Plus Gemcitabine and Cisplatin in Patients with Advanced Biliary Tract Cancer: A Real-World Retrospective Cohort Study

**DOI:** 10.3390/biomedicines13081915

**Published:** 2025-08-06

**Authors:** Eishin Kurihara, Satoru Kakizaki, Masashi Ijima, Takeshi Hatanaka, Norio Kubo, Yuhei Suzuki, Hidetoshi Yasuoka, Takashi Hoshino, Atsushi Naganuma, Noriyuki Tani, Yuichi Yamazaki, Toshio Uraoka

**Affiliations:** 1 Department of Gastroenterology, SUBARU Ota Memorial Hospital, Ota 3738585, Gunma, Japan; eishin.kurihara.dec.18@gmail.com (E.K.); ijima0711@gmail.com (M.I.); 2 Department of Gastroenterology, NHO Takasaki General Medical Center, Takasaki 3700829, Gunma, Japan; suy3k1yuhei@yahoo.co.jp (Y.S.); hybridrainbow916@yahoo.co.jp (H.Y.); fwnx6146@yahoo.co.jp (T.H.); naganuma2000@gmail.com (A.N.); 3 Department of Clinical Research, NHO Takasaki General Medical Center, Takasaki 3700829, Gunma, Japan; 4 Department of Gastroenterology, Gunma Saiseikai Maebashi Hospital, Maebashi 3710821, Gunma, Japan; hatanaka@qk9.so-net.ne.jp; 5 Department of Surgery, Gunma Saiseikai Maebashi Hospital, Maebashi 3710821, Gunma, Japan; nkubo503@gmail.com; 6 Department of Surgery, SUBARU Ota Memorial Hospital, Ota 3738585, Gunma, Japan; dr.wind@icloud.com; 7 Department of Gastroenterology and Hepatology, Gunma University Graduate School of Medicine, Maebashi 3718511, Gunma, Japan; yyuichi175@gunma-u.ac.jp (Y.Y.); uraoka@gunma-u.ac.jp (T.U.)

**Keywords:** durvalumab, gemcitabine, cisplatin, advanced biliary tract cancer, real-world data

## Abstract

**Background/Objectives**: The TOPAZ-1 phase III trial reported a survival benefit of using durvalumab, an anti-programmed death ligand 1 (anti-PD-L1) antibody, in combination with gemcitabine and cisplatin (GCD) treatment in patients with advanced biliary tract cancer. This retrospective study investigated the efficacy and safety of GCD treatment for advanced biliary tract cancer in real-world conditions. **Methods**: The study subjects were 52 patients with biliary tract cancer who received GCD therapy between January 2023 and May 2024. The observation parameters included the modified Glasgow Prognostic Score (mGPS), neutrophil–lymphocyte ratio (NLR), platelet–lymphocyte ratio (PLR), tumor markers (CEA, CA19-9), overall response rate (ORR), disease control rate (DCR), progression-free survival (PFS), overall survival (OS), and adverse events. **Results**: The cohort included 36 men and 16 women, with a median age of 73.0 years. There were 36 cases of cholangiocarcinoma (distal: 10, perihilar: 19, intrahepatic: 7), 13 cases of gallbladder cancer, and 3 cases of ampullary carcinoma. The stages were locally advanced in 30 cases and metastatic in 22 cases. Biliary drainage was performed in 30 cases. There were 38 cases receiving first-line therapy and 14 cases receiving second-line or later treatments. The median values at the start of GCD therapy were ALB 3.7 g/dL, CRP 0.39 mg/dL, NLR 2.4, PLR 162.5, CEA 4.8 ng/mL, and CA19-9 255.9 U/mL. The mGPS distribution was 0:23 cases, 1:18 cases, and 2:11 cases. The treatment outcomes were ORR 25.0% (CR 2 cases, PR 11 cases), DCR 78.8% (SD 28 cases, PD 10 cases, NE 1 case), median PFS 8.6 months, and median OS 13.9 months. The PLR was suggested to be useful for predicting PFS. A decrease in CEA at six weeks after the start of treatment was a significant predictor of PFS and OS. Gallbladder cancer had a significantly poorer prognosis compared to other cancers. The immune-related adverse events included hypothyroidism in two cases, cholangitis in one case, and colitis in one case. **Conclusions**: The ORR, DCR, and PFS were comparable to those in the TOPAZ-1 trial. Although limited by its retrospective design and small sample size, this study suggests that GCD therapy is an effective treatment regimen for unresectable biliary tract cancer in real-world clinical practice.

## 1. Introduction

Biliary tract cancer comprises a heterogeneous group of malignancies that include intrahepatic and extrahepatic cholangiocarcinoma, gallbladder cancer, and ampulla of Vater cancer [1]. Extrahepatic cholangiocarcinoma is further classified into perihilar and distal cholangiocarcinoma according to the anatomical location [1]. Despite recent advances in diagnostic technology, surgical resection, the only curative treatment, can be offered to up to one-quarter of patients [2]. Most patients are diagnosed at an advanced stage and are indicated for systemic chemotherapy, but the prognosis is poor [3]. Furthermore, the incidence of biliary tract cancer, intrahepatic cholangiocarcinoma in particular, is increasing globally, and it is problematic.

As a first-line treatment for advanced biliary tract cancer, the combination of gemcitabine and cisplatin (GC) is a standard regimen based on the positive results of two randomized trials [4,5]. The phase III trial ABC-02 showed improved progression-free survival (PFS) of 8.0 months for patients treated with GC compared to 5.0 months for patients treated with gemcitabine monotherapy, with an accompanying improvement in the median overall survival (OS) of 11.7 versus 8.1 months [4]. Biliary tract cancer exhibits immunogenic features, including expression of the immune checkpoint molecules programmed death ligand 1 (PD-L1) and cytotoxic T-lymphocyte-associated protein 4 (CTLA-4) in the tumor microenvironment [6,7]. Recently, the phase III TOPAZ-1 trial, with 685 patients, showed that the addition of immune checkpoint therapy using the anti-PD-L1 antibody durvalumab (D) in combination with GC increased patient survival [8,9,10]. The median overall survival (OS) for patients receiving the GCD treatment was 12.8 months compared to 11.5 months for those receiving GC plus placebo, with a reduction in the risk of death of 20% in favor of the experimental arm.

The results of the TOPAZ-1 trial led to United States Food and Drug Administration (FDA) and European Medicines Agency (EMA) approval of GCD therapy as the new first-line standard therapy for patients with unresectable or metastatic biliary tract cancer. In Japan, this combination therapy has been available since December 2022 in the real-world setting. Therefore, there is limited post-approval real-world data regarding its efficacy, safety, and predictive factors for a response in Japan.

We performed a multicenter retrospective analysis with the aim of investigating the efficacy and safety of this new first-line standard treatment in a real-world setting. The predictive factors, including systemic inflammation-based prognostic indicators, were also investigated.

## 2. Materials and Methods

**The Patients**: Between January 2023 and May 2024, a total of 52 Japanese patients with advanced biliary tract cancer who were receiving GCD treatment at NHO Takasaki General Medical Center, Gunma Saiseikai Maebashi Hospital, and SUBARU Ota Memorial Hospital were included and none were excluded from the current retrospective study. Patients were diagnosed with biliary tract cancer based on typical radiological or pathological findings. The authors retrospectively examined the medical records, collected patient characteristics, and analyzed the outcomes, including the tumor response, OS, PFS, and adverse events (AEs).

**The combination of durvalumab plus gemcitabine and cisplatin (GCD) treatment and the assessment of the tumor response and AEs**: Patients were treated with durvalumab combined with gemcitabine and cisplatin administered intravenously in a 21-day cycle for up to eight cycles. Durvalumab (1500 mg) was administered on day 1 of each cycle in combination with gemcitabine (1000 mg/m^2^) and cisplatin (25 mg/m^2^), which were administered on days 1 and 8 of each cycle. After completion of up to eight cycles of gemcitabine and cisplatin, durvalumab monotherapy (1500 mg) was administered once every 4 weeks until clinical or imaging disease progression or until unacceptable toxicity.

Contrast-enhanced computed tomography (CT) was carried out every 6–12 weeks. The tumor response was evaluated based on the Response Evaluation Criteria in Solid Tumors (RECIST) version 1.1. The overall response rate (ORR) was defined as the sum of the complete response (CR) and partial response (PR). The disease control rate (DCR) was defined as the sum of the CR, PR, and stable disease (SD) rates. OS was defined as the period from the day of initial GCD treatment to the day of death or last visit. PFS was defined as the period from the day of initial GCD treatment to the day of the presence of disease progression or death. The performance status was evaluated by the Eastern Cooperative Oncology Group [11]. AEs related to GCD treatment were assessed using the Common Terminology Criteria for Adverse Events version 5.0 [12]. The serum levels of CEA and CA19-9 were measured at baseline and every 6-week interval. The ratio of tumor markers before administration to 6 weeks after the start of administration was less than 1, which was defined as a decrease group, and 1 or more, which was defined as an increase group. The modified Glasgow Prognostic Score (mGPS) was determined as described previously [13,14]. Patients were stratified into 3 mGPS groups: mGPS 0 (CRP ≤ 0.5 and albumin ≥ 3.5 g/dL), mGPS 1 (CRP > 0.5 mg/L or albumin < 3.5 g/dL), and mGPS 2 (CRP > 0.5 mg/L and albumin < 3.5 g/dL). The neutrophil-to-lymphocyte ratio (NLR), platelet-to-lymphocyte ratio (PLR), and CRP–Alb ratio were calculated. The prognostic nutritional index (PNI) was calculated as follows: PNI = [10 × serum albumin (g/dL)] + [0.005 × total lymphocyte count (/mm^3^)]. These values were calculated immediately before the administration of GCD treatment.

**Statistical analyses:** Categorical variables are presented as numbers and percentages, and continuous variables are presented as the median (interquartile range [IQR]). Differences between groups were analyzed using Fisher’s exact test and the Mann–Whitney U test when a significant difference was obtained using the Kruskal–Wallis test. The prognosis was assessed using a Cox hazard analysis, the Kaplan–Meier method, and a log-rank test. All the statistical analyses were undertaken using EZR version 1.61 (Saitama Medical Center, Jichi Medical University, Saitama, Japan) [15]. A *p*-value of <0.05 was considered to indicate statistical significance.

## 3. Results

The patient characteristics are summarized in Table 1. The median age of all the patients was 73.0 (IQR 67.8–77.3) years old, and there were 36 (69.2%) men. The median body mass index (BMI) before GCD treatment was 21.9 (IQR: 19.7–23.7) kg/m^2^. There were 36 cases of cholangiocarcinoma (distal: 10, perihilar: 19, intrahepatic: 7), 13 cases of gallbladder cancer, and 3 cases of ampullary carcinoma. Concerning tumor factors, 30 patients (57.7%) had local advanced disease, and 22 patients (42.3%) had metastases. There were 20 patients (38.5%) with postoperative recurrence. Biliary drainage was performed in 30 cases. The ECOG-PS distribution was 0, 1, and 2 in 43 (82.7%), 7 (13.5%), and 3 patients (5.8%), respectively. There were 38 cases (73.1%) receiving first-line therapy and 14 cases receiving second-line or later treatments. A history of first-line treatment with gemcitabine plus cisplatin (GC), GC plus S-1 (GCS), and gemcitabine was noted in 12 (23.1%), 1 (1.9%), and 1 patient (1.6%), respectively. The serum levels of CEA and CA19-9 at baseline were 4.8 (IQR 2.6–10.5) ng/mL and 255.9 (IQR 40.8–1014.3) U/mL, respectively.

### 3.1. The OS and PFS

**Figure 1:**
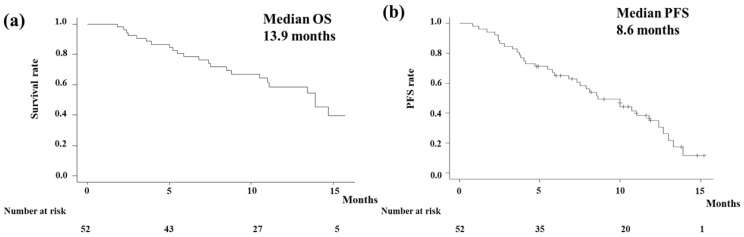
(**a**) The overall survival (OS) with durvalumab plus gemcitabine and cisplatin (GCD). The Kaplan–Meier curve showed that the median OS of all patients was 13.9 months. (**b**) The progression-free survival (PFS) with GCD. The median PFS in all patients was estimated to be 8.6 months.

**Figure 2:**
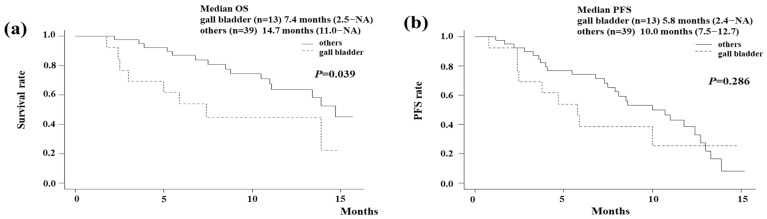
(**a**) The overall survival (OS) with durvalumab plus gemcitabine and cisplatin (GCD) according to the primary tumor type. The Kaplan–Meier curve showed that the median OS of gallbladder cancer was 7.4 months (95% CI 2.5–NA months). On the other hand, that of other cancers was 14.7 months (95% CI 11.0–NA months). Gallbladder cancer had a significantly poorer OS compared to other cancers (*p* = 0.039). (**b**) The progression-free survival (PFS) with GCD according to the primary tumor type. The Kaplan–Meier curve showed that the median PFS of gallbladder cancer was 5.8 months (95% CI 2.4–NA months). On the other hand, that of other cancers was 10.0 months (95% CI 7.5–12.7 months). Although it did not reach statistical significance, gallbladder cancer tended to have a poorer PFS compared to other cancers.

In the analysis of OS, events occurred in 23 patients (44.2%), with a median follow-up period of 10.1 months (95% confidence interval [CI] 8.9–11.3 months). The Kaplan–Meier curve showed that the median OS of all the patients was 13.9 months (95% CI 10.5-NA months; Figure 1a). Figure 1b shows the PFS of all the patients. Events were observed in 33 patients (63.5%) in the analysis of PFS. The median PFS in all the patients was estimated to be 8.6 (95% CI 6.8–11.8) months. According to the primary tumor type, OS and PFS are shown in Figure 2. The Kaplan–Meier curve showed that the median OS of gallbladder cancer was 7.4 months (95% CI 2.5–NA months; Figure 2a). On the other hand, that of other cancers was 14.7 months (95% CI 11.0–NA months). The median OS of extrahepatic cholangiocarcinoma (distal/perihilar), intrahepatic cholangiocarcinoma, and ampullary carcinoma were 13.4/14.7, NA, and 7.4 months, respectively. Gallbladder cancer had significantly poorer OS compared to other cancers (I = 0.039). The Kaplan–Meier curve showed that the median PFS of gallbladder cancer was 5.8 months (95% CI 2.4–NA months; Figure 2b). On the other hand, that of other cancers was 10.0 months (95% CI 7.5–12.7 months). Although it did not reach statistical significance, gallbladder cancer tended to have poorer PFS compared to other cancers.

**Figure 3:**
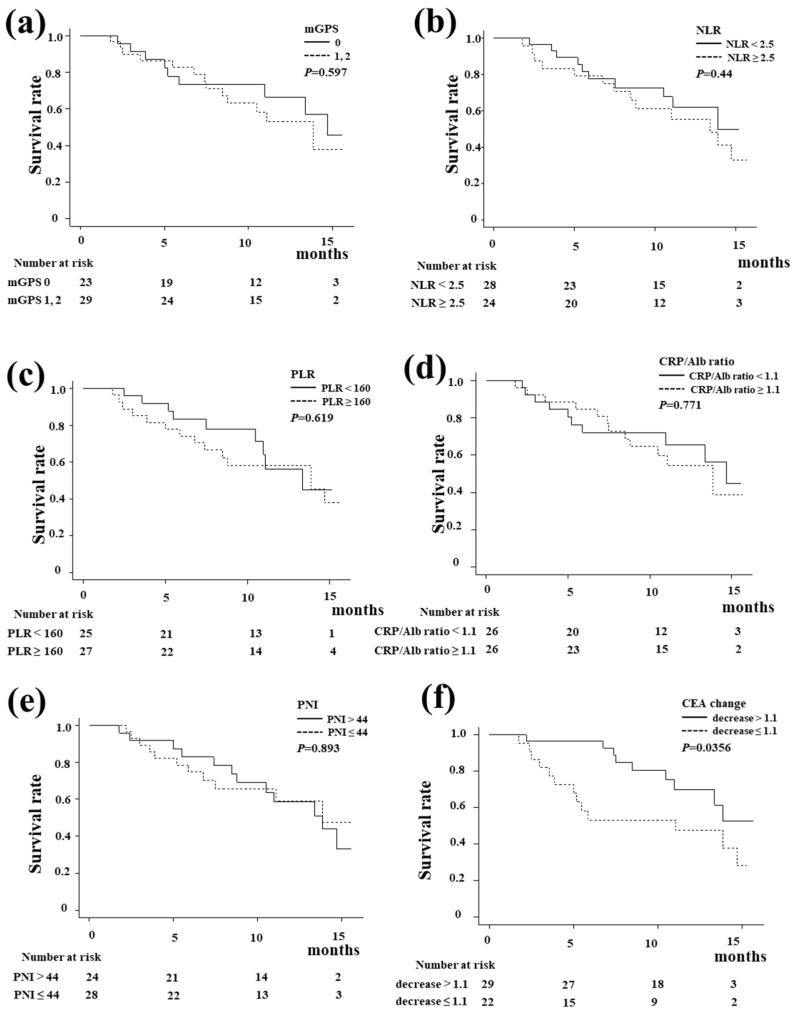
The overall survival (OS) with durvalumab plus gemcitabine and cisplatin (GCD) according to the systemic inflammation-based prognostic indicators and tumor marker change. (**a**) The modified Glasgow Prognostic Score (mGPS). (**b**) The neutrophil-to-lymphocyte ratio (NLR). (**c**) The platelet-to-lymphocyte ratio (PLR). (**d**) CRP–Alb ratio. (**e**) The prognostic nutritional index (PNI). (**f**) The CEA change. There were no significant differences in OS regarding the mGPS score, NLR, PLR, CRP–Alb ratio, or PNI. A decrease in CEA at six weeks after the start of treatment was a significant predictor of OS (*p* = 0.0356).

**Figure 4:**
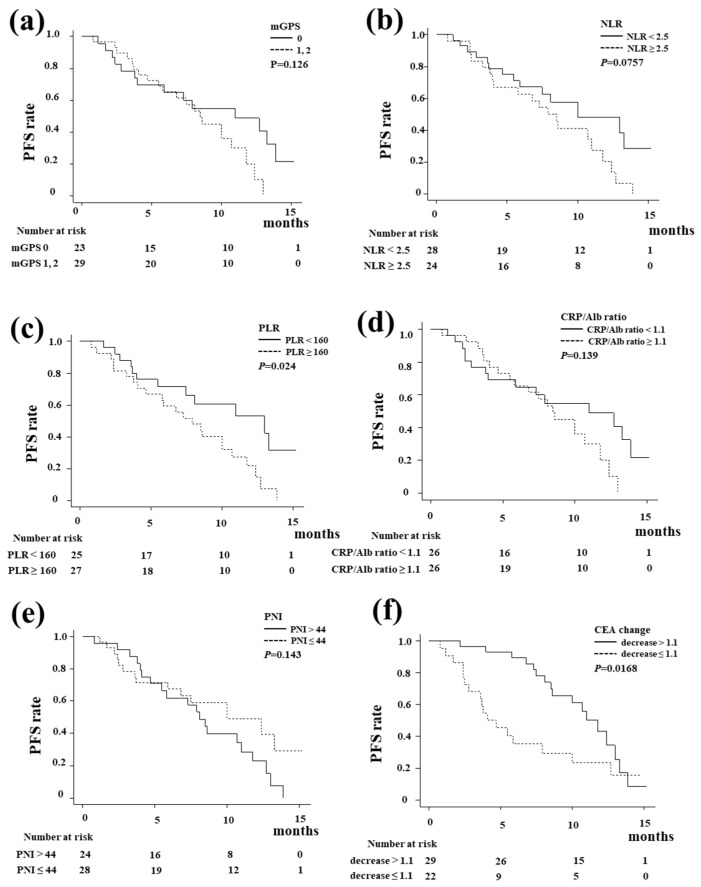
The progression-free survival (PFS) with durvalumab plus gemcitabine and cisplatin (GCD) according to the systemic inflammation-based prognostic indicators and tumor marker change. (**a**) The modified Glasgow Prognostic Score (mGPS). (**b**) The neutrophil-to-lymphocyte ratio (NLR). (**c**) The platelet-to-lymphocyte ratio (PLR). (**d**) CRP–Alb ratio. (**e**) The prognostic nutritional index (PNI). (**f**) The CEA change. There were no significant differences in the PFS regarding the mGPS score, NLR, CRP–Alb ratio, or PNI. The PLR was significantly associated with the PFS (*p* = 0.024). A decrease in CEA at six weeks after the start of treatment was also a significant predictor of PFS (*p* = 0.0168).

Figure 3 shows the Kaplan–Meier curve of OS according to the systemic inflammation-based prognostic indicators and tumor marker change. There were no significant differences in OS regarding the mGPS score, NLR, PLR, CRP–Alb ratio, or PNI. A decrease in CEA at six weeks after the start of treatment was a significant predictor of OS (*p* = 0.0356). Figure 4 shows the Kaplan–Meier curve of PFS. The PLR (Figure 4c, *p* = 0.024) was significantly associated with PFS, whereas the mGPS, NLR, CRP–Alb ratio, and PNI did not influence PFS. A decrease in CEA at six weeks after the start of treatment was also a significant predictor of PFS (*p* = 0.0168).

### 3.2. The ORR and DCR

The results associated with the tumor response are shown in Table 2. According to the RECIST, 2 patients (3.8%) had CR, 11 patients (21.2%) had PR, 28 (53.8%) had SD, 10 (19.2%) had PD, and 1 patient (1.9%) was not evaluable (NE). Thus, the ORR and DCR in all the patients were calculated to be 25.0% (13/52) and 78.8% (41/52), respectively.

### 3.3. AEs

The AEs during GCD treatment are summarized in Table 3. The adverse events were manageable, although neutropenia (42.3%), anemia (25.0%), thrombocytopenia (13.5%), and gastrointestinal symptoms such as dysgeusia, loss of appetite, nausea, and constipation were observed. The most frequent grade ≥ 3 AE was neutropenia, reported in 28.8% of patients, followed by anemia and general fatigue. The immune-related adverse events included hypothyroidism in two cases, cholangitis in one case, and colitis in one case. These four patients with immune-related adverse events were all grade ≥ 3.

## 4. Discussion

The main finding of the present study was that GCD therapy was considered to be an effective regimen for unresectable advanced biliary tract cancer even in clinical practice. The median PFS and OS of our cohort were 8.6 and 13.9 months, respectively. Those of the TOPAZ-1 trial were 7.2 months for PFS and 12.8 months for OS, respectively. In other words, this study demonstrated that the results in a real-world setting can be comparable to those obtained in randomized controlled trials, which enrolled patients with better clinical characteristics. The treatment outcomes of this study were ORR 25.0% and DCR 78.8%. Those of the TOPAZ-1 trial were 26.7% for ORR and 85.3% for DCR. We were also able to demonstrate that the ORR and DCR results of this real-world study were comparable to those of the TOPAZ-1 trial [8].

Biliary tract cancers are aggressive tumors known to be characterized by a scarce response to treatment. The ABC-02 trial reported an ORR of 26.1% and a DCR of 81.4% in patients with biliary tract cancer who were receiving cisplatin plus gemcitabine [4,5], and GC treatment contributed as a first-line regimen for more than 10 years. Immune checkpoint inhibitors have been used in various types of cancer, and their effectiveness has been reported. The first immune checkpoint inhibitor to be used in biliary tract cancer was the TOPAZ-1 regimen [8,9,10], in which durvalumab was added to the standard GC treatment. The benefit of combining chemotherapy and immunotherapy in biliary tract cancers has recently been confirmed by the KEYNOTE-966 phase III study of pembrolizumab plus GC [16].

The TOPAZ-1 study did not include ampullary carcinoma, and there were no data for ampullary carcinoma. This study included three cases of ampullary carcinoma. We could not confirm the result of the treatment effect due to the small number of cases. Further investigation should be performed by adding additional cases in the future. In this study, we showed that gallbladder cancer had a significantly poorer prognosis compared to other cancers.

For predicting the treatment response, a decrease in CEA at six weeks after the start of treatment was a significant predictor of PFS and OS in this study. In other words, it is possible to predict the therapeutic effect at a relatively early stage of treatment, which is clinically useful. The PLR showed a significant difference as a predictive factor for PFS in the present study. Inflammation has been considered to play an essential role in cancer progression. A number of inflammation-based prognostic factors have been developed, including the GPS, mGPS, PLR, NLR, CRP–Alb ratio, and PNI [13,14,17,18,19,20]. We evaluated these inflammation-based prognostic factors in this study. The PLR only showed a significant difference in the present study. A high PLR, indicating systemic inflammation and potential immunosuppression, is often associated with poorer outcomes in patients treated with ICIs [21]. Lymphocytes and platelets play pivotal roles in the systemic inflammatory response, demonstrating crucial functions in tumor development, infiltration, and metastasis [22]. Tumor cells employ various mechanisms to activate platelets, leading to the direct release of factors such as IL-1, thrombin, and endothelin, thereby promoting tumor angiogenesis and enhancing tumor migration and dissemination [23]. A reduced lymphocyte count could result in an inadequate immune response, consequently exerting an adverse impact on the prognosis of patients with cancers [22]. Neutrophils inhibit an immune response by lymphocytes, natural killer cells, or activated T cells [24,25]. Although the PLR only showed a significant difference in the present study, not only the PLR but also the NLR might have shown statistical significance if the number of patients and the observation periods were increased.

In the TOPAZ-1 study, the most common adverse events were anemia (48.2%), nausea (40.2%), constipation (32.0%), and neutropenia (31.7%) in the GCD group [8]. In this study, neutropenia (42.3%), anemia (25.0%), and thrombocytopenia (13.5%) were similar. However, there were fewer referrals for gastrointestinal symptoms such as nausea, likely because these could be managed with antiemetics. In the TOPAZ-1 trial, the grade ≥ 3 AEs included anemia (23.7%), neutropenia (20.1%), and thrombocytopenia (4.7%) [8]. In this study, the grade ≥ 3 AEs included neutropenia (28.8%) and anemia (5.8%). Neutropenia was common, but anemia and thrombocytopenia were less common compared to TOPAZ-1. The immune-related adverse events included hypothyroidism in two cases, cholangitis in one case, and colitis in one case. In the TOPAZ-1 trial, any immune-mediated adverse events were observed in 43/338 cases (12.7%), and hypothyroid events (5.9%) were most common, followed by dermatitis (3.6%), hepatic events (1.2%), and adrenal insufficiency (1.2%) [8].

The current study had several limitations. First, this was a retrospective study, and the number of patients was relatively small. Although this is sufficient in terms of reporting early real-world clinical data, the examination of predictive factors is a future task as more detailed markers may be identified if the number of cases increases.

## 5. Conclusions

Our data mostly confirmed the results achieved in the TOPAZ-1 trial in terms of PFS, ORR, and safety, supporting the use of this combination in clinical practice. The PLR and a decrease in CEA at six weeks were suggested to be potentially useful for predicting the response. GCD therapy is an effective regimen for unresectable biliary tract cancer in real-world clinical practice.

## Figures and Tables

**Table 1:** Patients’ baseline characteristics.

Characteristics		Number (%) or Median (IQR)
Patients, *n* (%)		52 (100)
Sex, *n* (%)	Male	36 (69.2)
	Female	16 (30.8)
Age, years (IQR)		73.0 (67.8–77.3)
BMI, kg/m^2^ (IQR)		21.9 (19.7–23.7)
Primary tumor type, *n* (%)	Intrahepatic cholangiocarcinoma	7 (13.5)
	Extrahepatic cholangiocarcinoma (distal/perihilar)	10 (19.2)/19 (36.5)
	Gall bladder	13 (25.0)
	Ampullary carcinoma	3 (5.8)
Cancer stage, *n* (%)	Local/locally advanced	30 (57.7)
	Metastatic	22 (42.3)
Performance status	0/1/2	43 (82.7)/7 (13.5)/2 (3.8)
Alb, g/dL (IQR)		3.7 (3.4–4.0)
CRP (IQR)		0.39 (0.17–2.08)
mGPS (%)	0/1/2	23 (44.2)/18 (34.6)/11 (21.2)
NLR (IQR)		2.4 (1.7–3.9)
PLR (IQR)		162.5 (118.3–242.8)
CRP–Alb ratio (IQR)		0.12 (0.04–0.60)
PNI (IQR)		44.5 (40.3–48.1)
CEA (IQR)		4.8 (2.6–10.5)
CA19-9 (IQR)		255.9 (40.8–1014.3)
Line of therapy at which GCD was administered (%)	1st	38 (73.1)
	2nd	12 (23.1)
	3rd	2 (3.8)
First-line cancer therapy (%)	GCD	38 (73.1)
	GC	12 (23.1)
	GCS	1 (1.9)
	GEM	1 (1.9)

IQR, interquartile range; BMI, body mass index; Alb, albumin; mGPS, modified Glasgow Prognostic Score; NLR, neutrophil-to-lymphocyte ratio; PLR, platelet-to-lymphocyte ratio; PNI, prognostic nutritional index; GEM, gemcitabine.

**Table 2:** The overall response and disease control rates.

	All Patients (*n* = 52)
Complete response, *n* (%)	2 (3.8)
Partial response, *n* (%)	11 (21.2)
Stable disease, *n* (%)	28 (53.8)
Progressive disease, *n* (%)	10 (19.2)
Not evaluable, *n* (%)	1 (1.9)
Overall response rate (%)	13/52 (25.0%)
Disease control rate (%)	41/52 (78.8%)

**Table 3:** Adverse events during durvalumab plus gemcitabine and cisplatin treatment.

Toxicity	All	Grade 3/4
Neutropenia	22 (42.3)	15 (28.8)
Anemia	13 (25.0)	3 (5.8)
Thrombocytopenia	7 (13.5)	0 (0)
Appetite loss	5 (9.6)	0 (0)
General fatigue	4 (7.7)	2 (3.8)
Dysgeusia	3 (5.8)	0 (0)
Nausea	1 (1.9)	0 (0)
Cholangitis	2 (3.8)	1 (1.9) *
Colitis	1 (1.9)	1 (1.9) *
Constipation	2 (3.8)	0 (0)
Abdominal infection	1 (1.9)	1 (1.9)
Hypothyroidism	2 (3.8)	2 (3.8) *
Eruption	2 (3.8)	0 (0)
AST increased	6 (11.5)	1 (1.9)
ALT increased	5 (9.6)	1 (1.9)
Creatinine increased	2 (3.8)	0 (0)
Hypersensitivity for cisplatin	2 (3.8)	1 (1.9)

* Immune-related adverse events included hypothyroidism in two cases, cholangitis in one case, and colitis in one case.

## Data Availability

All data generated or analyzed during this study are included in this article. Further inquiries can be directed to the corresponding author.

## Abbreviations

The following abbreviations are used in this manuscript:

PD-L1	programmed death ligand 1
GCD	durvalumab plus gemcitabine and cisplatin
mGPS	modified Glasgow Prognostic Score
NLR	neutrophil–lymphocyte ratio
PLR	platelet–lymphocyte ratio
ORR	overall response rate
DCR	disease control rate
PFS	progression-free survival
OS	overall survival
CTLA-4	cytotoxic T-lymphocyte-associated protein 4
AEs	adverse events
RECIST	Response Evaluation Criteria in Solid Tumors
PNI	prognostic nutritional index
BMI	body mass index
